# Decremental responses following repetitive nerve stimulation in spinal and bulbar muscular atrophy

**DOI:** 10.1016/j.cnp.2026.06.011

**Published:** 2026-06-26

**Authors:** Keisuke Tachiyama, Masahiro Nakamori, Yuki Fujii, Keiichi Hokkoku, Dai Agari, Shoji Hemmi, Eiichi Nomura, Yuki Hatanaka, Katsumi Kurokawa, Masahiro Sonoo, Hirofumi Maruyama

**Affiliations:** a Department of Clinical Neuroscience and Therapeutics, Hiroshima University Graduate School of Biomedical and Health Sciences, Japan; b Department of Neurology, Hiroshima City Hiroshima Citizens Hospital, Hiroshima, Japan; c Department of Neurology, Kawasaki Medical School, Kurashiki, Japan; d Department of General Internal Medicine, Kawasaki Medical School, Kurashiki, Japan; e Department of Neurology, Teikyo University School of Medicine, Tokyo, Japan; f Department of Orthoptics, Faculty of Medical Technology, Teikyo University, Tokyo, Japan

**Keywords:** Repetitive nerve stimulation, Spinal and bulbar muscular atrophy, Decremental response

## Abstract

**Objective:**

The presence of decremental responses following repetitive nerve stimulation (RNS) in amyotrophic lateral sclerosis (ALS) is well established. However, in spinal and bulbar muscular atrophy (SBMA), a rare X-linked recessive lower motor neuron disease, the incidence and distribution of decremental responses across different muscles have not been thoroughly investigated.

**Methods:**

Patients with SBMA were retrospectively identified in our database. RNS at a frequency of 3 Hz was performed on five muscles: the abductor pollicis brevis (APB), abductor digiti minimi (ADM), upper trapezius, deltoid, and facial muscles (frontalis or nasalis).

**Results:**

A total of forty patients were identified. A significant (> 5%) decremental response in at least one muscle was observed in all patients. It was observed more frequently in proximal muscles than in distal muscles: deltoid (86%), trapezius (70%), facial muscles (44%), APB (37%) and ADM (25%). The magnitude of the decremental response in the deltoid was significantly higher than that in the other muscles.

**Conclusions:**

Our results demonstrated that decremental responses were frequently observed in patients with SBMA, with a distribution pattern similar to that in ALS. The fact that the decremental responses are observed in SBMA having an extremely chronic course would be relevant for the pathophysiological mechanism of the decremental response.

**Significance:**

The RNS findings provide valuable insights into the pathological mechanisms of SBMA and may contribute to the development of future treatments.

## Highlights

• Significant decremental responses were observed in spinal and bulbar muscular atrophy.
• Proximal muscles showed a predominant pattern of decrement.
• The deltoid exhibited the largest decrement magnitude.

## 1 Introduction

Repetitive nerve stimulation (RNS) is widely used to assess the function of the neuromuscular junction. A decremental response following RNS is well known to occur in those with myasthenia gravis (MG). The presence of decremental responses following RNS in individuals with amyotrophic lateral sclerosis (ALS) has also been documented (Mulder et al., 1959; Bernstein and Antel, 1981; Killian et al., 1994). Recently, we studied the frequency and magnitude of decremental responses in the individual muscles of those with ALS; we found that significant (> 5%) decremental responses were most frequently observed in proximal muscles, such as the deltoid and trapezius (Iwanami et al., 2011). The decrement observed in those with ALS is often thought to be caused by immature reinnervating axonal branches (Bennett et al., 1973; Stålberg et al., 1975). Thus, a decrement is less likely to be observed in spinal and bulbar muscular atrophy (SBMA), which has a more chronic course.

However, previous reports on decremental responses in SBMA have been limited by small sample sizes and have primarily focused on a restricted number of muscles, most commonly the trapezius (Inoue et al., 2009; Kim et al., 2013). As a result, the overall distribution pattern of decremental responses across different muscle groups has not been systematically characterized. In particular, the deltoid muscle, which has been shown to be highly sensitive for detecting decremental responses in ALS, has not been sufficiently investigated in SBMA. Therefore, in this study, we aimed to systematically evaluate the frequency, magnitude, and distribution pattern of decremental responses across multiple muscles in a larger cohort of patients with SBMA.

## 2 Methods

### 2.1 Participants

We searched the electromyography (EMG) and in-patient databases of Hiroshima University Hospital, Kawasaki Medical School Hospital, Teikyo University Hospital, and their related institutions from January 2008 to January 2025 using the keyword of SBMA. Medical records and EMG reports of extracted patients were retrospectively reviewed. Consecutive patients who underwent the examination were included.

**Fig. 1:**
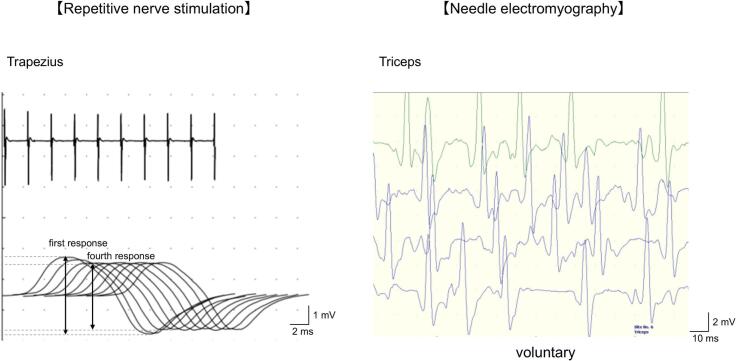
Repetitive nerve stimulation (trapezius) and needle electromyography (triceps) findings in a patient with spinal and bulbar muscular atrophy.

The inclusion criteria were: (1) RNS was performed by board-certified neurologists; (2) RNS data of the following five muscles were available: abductor pollicis brevis (APB), abductor digiti minimi (ADM), upper trapezius (Trap), deltoid (Del), and one of the following facial muscles, either frontalis (Front) or nasalis (Nas); and (3) the diagnosis of SBMA was confirmed by identifying CAG repeat expansions through genetic testing. In cases where genetic testing had not been performed, a diagnosis of SBMA was made based on a combination of clinical features, including a positive family history (a male relative diagnosed with SBMA), slowly progressive lower motor neuron involvement, and characteristic neurological findings. Only patients with clinically consistent features of SBMA were included in the analysis. All patients underwent needle EMG of limb muscles to assess for reduced recruitment and the presence of giant motor unit potentials during voluntary contraction (representative findings are shown in Fig. 1).

Clinical data collected from patients with SBMA included age, disease duration, availability of genetic testing, number of CAG repeats, serum creatine kinase (CK) levels, initial symptoms and the modified Rankin Scale (mRS) score at the time of examination. This study was approved by the Ethics Committee of Hiroshima University Hospital (Approval number: E2023–0200).

### 2.2 Repetitive nerve stimulation

All examinations were conducted using a standard electromyography machine (Neuropack 2200 or 2300 series; Nihon-Koden Co, Tokyo, Japan). Bipolar surface-stimulating electrodes were used for stimulation. The compound muscle action potential (CMAP) from the examined muscles was recorded using Ag-AgCl cup electrodes. The bandpass filter was set between 10 Hz and 3 kHz (−3 dB). For distal muscles, skin temperature was measured at the center of the palm and maintained at 33 °C or higher throughout the examination. A square wave pulse of 0.2-ms duration was delivered. RNS was performed with 10 pulses delivered at a frequency of 3 Hz in the APB, ADM and Trap muscles. Six pulses were performed instead when the stimulation was rather painful, such as for the Del or facial muscles. The magnitude of the decremental response was determined by measuring the decrement in the peak-to-peak amplitude of the CMAP between the first and fourth responses (a representative waveform is shown in Fig. 1). Although a decremental response exceeding 10% is commonly regarded as abnormal, previous reports have stated that a decrement greater than 5% can be considered abnormal when the technique is reliable (Keesey, 1989; Sanders, 1993). In an analysis of 20 normal control subjects, the maximal decrement observed across all six tested muscles (APB, ADM, extensor digitorum communis, Trap, Del, Nas) was 3%, and the upper limit based on the 95% prediction interval was 4.1% for the Trap, which showed the highest value (Iwanami et al., 2011). Accordingly, we considered 5% as the upper limit of normal. Subsequent studies have also employed this criterion (Hatanaka et al., 2017; Takahashi et al., 2024), and so we judged a decremental response exceeding 5% to be abnormal. Abnormalities were categorized into two groups: those with decrements exceeding 5% (> 5%) and those exceeding 10% (> 10%).

A so-called U-shape (Sanders, 1993; Shapiro and Preston, 2003), in which the response amplitude increases again after reaching the bottom response during RNS, was evaluated by calculating the difference in the magnitude of the decrement between the 10th response and the bottom response, based on previous reports (Iwanami et al., 2011). This parameter was evaluated in the ADM and the Trap, in both of which 10 stimulations were routinely applied and a decremental response exceeding 5% was observed. We performed this analysis only when the responses were clearly delineated and could be evaluated up to the 10th waveform.

### 2.3 Statistical analyses

We evaluated two RNS parameters: (1) the frequency of positive decremental responses and (2) the magnitude of the decremental response. Statistical analyses were performed using clinical data and RNS parameters with JMP Pro 18 software (JMP Statistical Discovery LLC, Cary, NC, USA). To compare the magnitude of decremental responses in each muscle, the Kruskal–Wallis test was employed to determine significant differences, if the data did not follow a normal distribution by the Shapiro–Wilk test. For multiple comparisons, the Steel–Dwass test was applied. The Pearson correlation coefficient was used to assess the relationship between the magnitude of the decremental response in each muscle and clinical data; if the data were not normally distributed by the Shapiro–Wilk test, the Spearman's rank correlation coefficient was applied. A *p*-value of less than 0.05 was considered statistically significant.

## 3 Results

### 3.1 Patients

In total, 40 patients were identified. The number of muscles examined was as follows: APB, *n* = 35; ADM, *n* = 32; Trap, *n* = 37; Del, n = 35; facial muscles, n = 32 (Nas, *n* = 21; Front, *n* = 11). The RNS findings and clinical features of patients with SBMA are summarized in Table 1. The genetic testing was conducted in 27 patients. About half of the initial symptoms were lower extremity symptoms, with many patients using a cane for ambulation, but none required a gastrostomy or respiratory support.

**Table 1:** Clinical features.

Total	*n* = 40
Institution	
Hiroshima University Hospital, n(%)	4 (10)
Hiroshima City Hiroshima Citizens Hospital, n(%)	5 (13)
Kawasaki Medical School Hospital, n(%)	4 (10)
Teikyo University Hospital, n(%)	18 (45)
Related institutions, n(%)	9 (23)
Age, years	58.2 ± 12.2
Disease duration, years	6.9 ± 6.9

Initial symptoms	
Bulbar, n(%)	8 (20)
Upper limb, n(%)	11 (28)
Lower limb, n(%)	21 (53)
	
mRS	
mRS 1, n(%)	18 (45)
mRS 2, n(%)	11 (28)
mRS 3, n(%)	7 (18)
mRS 4, n(%)	4 (10)
Serum CK (IU/L)⁎	668.5 ± 458.2
CAG repeat§	46.8 ± 4.0

mRS, modified Rankin Scale; CK, creatine kinase.

⁎ The number of cases in which CK testing was performed was 30.

§ The number of cases in which genetic testing was performed was 27.

### 3.2 RNS results

**Fig. 2:**
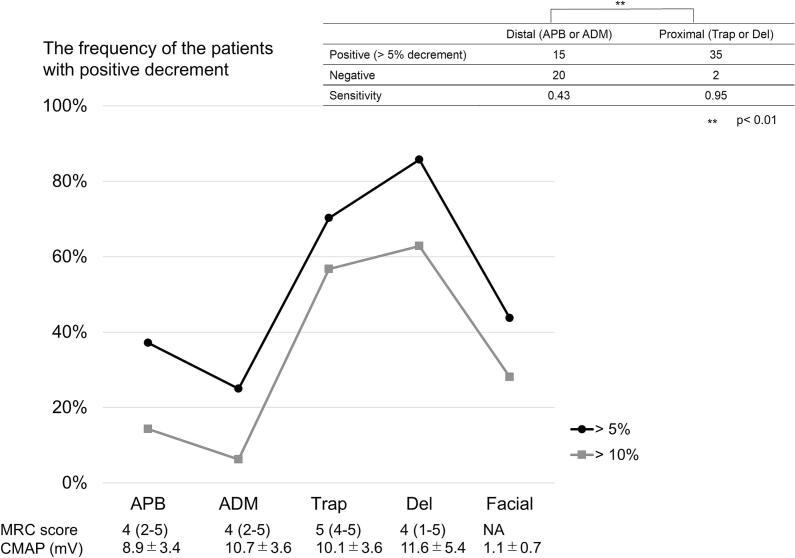
The frequency of the decremental responses in each muscle. The number of muscles examined was as follows: APB, *n* = 35; ADM, *n* = 32; Trap, *n* = 37; Del, n = 35; Facial, n = 32 (nasalis, *n* = 21; frontalis, *n* = 11). The median MRC score (minimum–maximum) for each muscle is shown below the corresponding muscle name. Below that, the mean CMAP amplitude of the first response in RNS is listed. APB, Abductor pollicis brevis; ADM, Abductor digiti minimi; Trap, Trapezius; Del, Deltoid; Facial, Facial muscle; NA, not applicable.

The percentage of patients showing significant decremental responses for each muscle is visualized in Fig. 2. A decremental response exceeding 5% was most frequently observed in the Del (86%), followed by the Trap (70%). Similarly, a decremental response greater than 10% was most frequently seen in the Del (63%), followed by the Trap (57%). In general, a decremental response was more frequently observed in proximal muscles than in distal muscles (APB and ADM). In almost all of the patients with SBMA (95%), a decrement was observed in either the deltoid or the trapezius muscle.

**Fig. 3:**
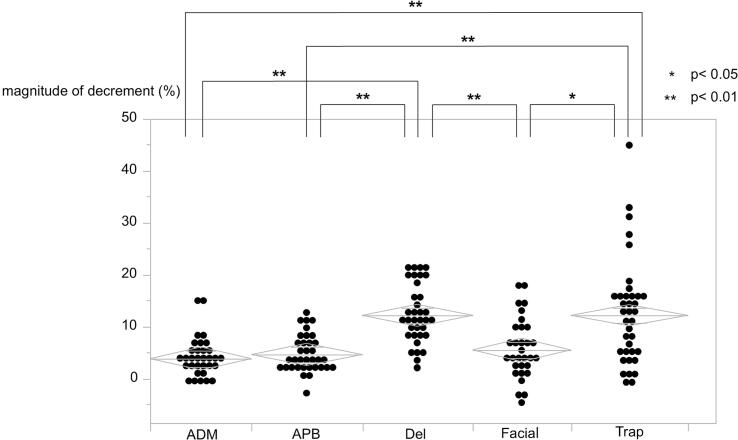
The comparison of the magnitude of the decremental response in each muscle. The Steel–Dwass test was applied for multiple comparisons to compare the degree of decrement among different muscles. The number of muscles analyzed was as follows: APB, n = 35; ADM, n = 32; Trap, n = 37; Del, n = 35; Facial, n = 32 (nasalis, n = 21; frontalis, n = 11). APB, Abductor pollicis brevis; ADM, Abductor digiti minimi; Trap, Trapezius; Del, Deltoid; Facial, Facial muscle.

Next, we investigated whether the magnitude of the decrement was also significantly greater in proximal muscles, consistent with the higher frequency observed in these muscles. The magnitude of the decremental response across muscles was analyzed using the Kruskal–Wallis test, which revealed significant differences (*p* < 0.0001). To further investigate which muscles showed significant differences, a Steel–Dwass test was performed. The Del exhibited a significantly higher decremental response than all other muscles except the Trap (Fig. 3). Furthermore, when comparing the magnitude of the decrement between distal muscles (APB and ADM) and proximal muscles (Trap and Del), the proximal muscles showed a significantly greater decrement than the distal muscles. We examined the correlations between the magnitude of the decrement in each muscle and the clinical information shown in Table 1, but no correlations were observed (Table 2). In addition to the clinical findings, we also investigated the correlations between the Medical Research Council score, the peak-to-peak amplitude of the CMAP of the first response in RNS, and the magnitude of the decrement; however, no clear correlation was observed (Table 2).

**Table 2:** Correlation between the magnitude of decrement and clinical findings in each muscle (correlation coefficient: −1 < *r* < 1).

(r)	APB	ADM	Trap	Del	Facial
Age	0.07	0.14	0.14	0.02	0.16
Disease duration	−0.21	0.01	0.15	0.19	−0.36
Number of CAG repeats	−0.19	−0.23	−0.08	−0.02	−0.14
Serum CK	0.07	−0.13	−0.09	−0.20	−0.04
MRC score	−0.05	−0.03	−0.20	−0.002	
CMAP amplitude of the first response	−0.02	−0.04	−0.24	−0.02	−0.02
mRS	0.18	0.22	0.12	0.05	0.06

APB, Abductor pollicis brevis; ADM, Abductor digiti minimi; Trap, Trapezius; Del, Deltoid; Nas, Nasalis; Front, Frontalis; CK, creatine kinase; MRC, medical research council; CMAP, compound muscle action potential; mRS, modified Rankin Scale.

Regarding the U shape, the recovery of the amplitude of the 10th response from the bottom response was 1.2 ± 0.6% (*n* = 7) for the ADM and 1.8 ± 1.0% (*n* = 20) for the Trap.

## 4 Discussion

In this study, we examined the decremental responses following RNS in patients with SBMA and found that decrements were frequently observed in proximal muscles, such as the deltoid and trapezius. The magnitude of the decrement was highest in the deltoid muscle and, consistent with its frequency, was significantly greater in proximal muscles than in distal muscles. However, no correlation was found between the magnitude of the decrement and clinical data.

Decremental responses following RNS have also been reported in patients with ALS (Mulder et al., 1959; Bernstein and Antel, 1981; Killian et al., 1994), and some studies have described features in detail. In patients with ALS, decremental responses are more frequently observed in proximal muscles (Iwanami et al., 2011). In patients with SBMA, there have been a few reports on the presence of decremental responses. Previous reports primarily focused on the trapezius muscle and concluded that decremental responses were more frequently observed in proximal muscles than in distal muscles (Inoue et al., 2009; Kim et al., 2013). In this study, we extended these observations by systematically evaluating multiple muscles, including the deltoid, in a larger cohort. We found that almost all patients with SBMA exhibited an abnormal decremental response in proximal muscles (deltoid or trapezius), demonstrating a consistent distribution pattern with proximal predominance. Previous reports have shown that, in patients with ALS, the proportion exhibiting a decremental response exceeding 5% was 54% (26/48) in the APB, 35% (17/48) in the ADM, 76% (34/45) in the Del, and 71% (34/48) in the Trap, indicating a predominance of decremental responses in proximal muscles (Iwanami et al., 2011). In the present study, we examined a comparable number of muscles and similarly observed higher frequencies of decrement in proximal muscles: 37% (13/35) in the APB, 25% (8/32) in the ADM, 86% (30/35) in the Del, and 70% (26/37) in the Trap. Our study found that patients with SBMA exhibited a pattern of decremental responses similar to that in patients with ALS, particularly in terms of the frequency of responses in proximal muscles. This pattern-based characterization provides additional clinical insight beyond previous reports that examined limited muscle groups. Although RNS is not a highly sensitive or disease-specific test when considered in isolation, its diagnostic value may be enhanced when interpreted in the context of muscle-specific patterns. In patients with MG, proximal muscles are considered to be more susceptible to involvement both clinically and electrophysiologically, which has been attributed to a lower safety factor in these muscles (Krarup, 1983). In patients with ALS, the decremental response completed more rapidly in proximal muscles such as the Del than in distal muscles such as the APB. It has been suggested that this finding may be attributable to a lower safety factor in proximal muscles (Ueta et al., 2024). As in patients with ALS, patients with SBMA show a more pronounced decrement in proximal muscles, and a lower safety factor may be one of the contributing factors.

Regarding the pattern of decrement in RNS, the U-shape is considered characteristic of MG and has been reported as absent in patients with ALS (Killian et al., 1994; Shapiro and Preston, 2003). However, some reports have also described the presence of a U-shape in patients with ALS (Sanders, 1993). In a previous comparative analysis of the U-shape between patients with MG and those with ALS, the difference between the 10th response and the bottom response in RNS was evaluated. In the ADM, this difference was significantly greater in patients with MG (5.2 ± 2.0%, *n* = 9) than in those with ALS (2.2 ± 1.5%, *n* = 17). In contrast, in the Trap, no significant difference was observed between the two groups, with values of 1.6 ± 1.3% in the ALS group (*n* = 29) and 2.0 ± 1.0% in the MG group (*n* = 26) (Iwanami et al., 2010). In our study, the values were 1.2 ± 0.6% for the ADM (*n* = 7) and 1.8 ± 1.0% for the Trap (*n* = 20) in patients with SBMA. These results were closer to those previously reported in patients with ALS, although we were unable to perform a direct comparison among patients with SBMA, ALS, and MG. These findings suggest that, even in terms of the U-shape, SBMA shares physiological characteristics with ALS.

It is supposed that there are many immature sprouts due to ongoing reinnervation in rapidly progressive neurogenic disorders like ALS, which would cause decremental responses following RNS. Therefore, the magnitude of the decremental response is expected to correlate with the speed of progression in ALS pathology. However, our study documented no correlation between the decremental response and the speed of progression. Based on these results, we postulated that not only immature endplates but also mature endplates following maximal reinnervation may also contribute to the decremental response in RNS (Fujii et al., 2025). This hypothesis can explain why decremental responses are observed in SBMA, a much more chronic disorder than ALS.

The present study has a few limitations. First, sufficient cases were not identified, due to the rarity of the disease and a control group was not included in this study. A larger sample size may increase the likelihood of identifying correlations with clinical findings. Second, genetic confirmation was not available in all patients, which may have introduced diagnostic uncertainty; however, we applied strict clinical inclusion criteria to minimize this risk. Third, in the assessment of facial muscle, the selection of the target muscle varied across institutions, with either the nasalis or the frontalis muscle examined. Fourth, some patients were undergoing treatment with leuprorelin during the evaluation. We did not compare the magnitude of decrement before and after treatment, which presents an opportunity for further investigation in future studies.

## 5 Conclusions

We observed a decremental response following RNS in patients with SBMA, which follows a more chronic course than ALS. As in patients with ALS, the assessment of proximal muscles such as the trapezius and deltoid is important. The mechanism underlying the decremental responses in patients with SBMA remains unclear, but it may be due to presynaptic abnormalities at the neuromuscular junction, similar to ALS. The precise mechanism underlying the decremental responses in patients with SBMA remains unclear; however, presynaptic abnormalities at the neuromuscular junction may be involved, similar to ALS. Electrophysiological findings in SBMA, including our RNS findings, may contribute to elucidating its underlying pathophysiology.

## Authors contribution

KT, MN, and KK conceptualized the study. KT, SH, KK and MS examined the patients. KT collected data from our laboratory. YF, KH, DA, EN, and SH collected data from other laboratories. KT prepared the initial draft. MN, KK and MS revised and drafted the manuscript. EN, YH, HM, and MS supervised this study. All the authors reviewed and approved the final version of the manuscript.

## Ethics approval and consent to participate

Ethical approval was obtained from each institution's ethics committee. The examinations were conducted as part of routine medical practice, and patients were provided with informed consent, including the option to decline participation in the study. All procedures involving human participants were performed in accordance with the 1964 Declaration of Helsinki and its later amendments or comparable ethical standards.

## Funding

This work was supported by Grants-in-Aid for Scientific Research [grant numbers 25K02979, 23H02827].

## Declaration of competing interest

The authors declare that they have no known competing financial interests or personal relationships that could have appeared to influence the work reported in this paper.

## Data Availability

All data related to the patients and the study are available upon reasonable request from the corresponding author.
